# Combined evaluation of coronary artery disease and high-sensitivity cardiac troponin T for prediction of adverse events in patients with hypertrophic cardiomyopathy

**DOI:** 10.1186/s12872-021-02135-x

**Published:** 2021-07-03

**Authors:** Hang Liao, Huay Cheem Tan, Ziqiong Wang, Xiaoping Chen, Yong He, Sen He

**Affiliations:** 1 Department of Cardiology, West China Hospital of Sichuan University, Chengdu, 610041 China; 2 Department of Cardiology, National University Heart Centre, Singapore, Singapore

**Keywords:** Hypertrophic cardiomyopathy, High-sensitivity cardiac troponin T, Coronary artery disease, Major adverse cardiovascular events

## Abstract

**Background:**

This study was performed to investigate the clinical significance of combined evaluation of both coronary artery disease (CAD) and high-sensitivity cardiac troponin T (hs-cTnT) for prediction of major adverse cardiovascular events (MACEs) in patients with hypertrophic cardiomyopathy (HCM).

**Methods:**

We performed clinical evaluations, including coronary artery imaging and hs-cTnT measurement, in 162 patients with HCM.

**Results:**

The patients were followed up for a median period of 3.7 years (interquartile range 2.4–5.6 years; total of 632.3 person-years [PYs]), during which time MACEs occurred in 24 (14.8%) patients. The incidence of MACEs was 6.4 and 2.7 per 100 PYs for patients with CAD and normal coronary arteries, respectively; similarly, the incidence was 5.8 and 2.1 per 100 PYs in patients with an elevated hs-cTnT concentration (> 14.0 ng/L) and a normal hs-cTnT concentration, respectively. The multivariate analysis suggested that CAD and an elevated hs-cTnT concentration tended to be positively associated with MACEs. When the groups were allocated according to these two markers, the patients were divided into four groups, which further improved the predictive values. The incidence of MACEs was 10.4 per 100 PYs in the CAD and elevated hs-cTnT group, which was much higher than the incidence in all other groups (range, 2.0–3.5 per 100 PYs). With the normal coronary arteries and normal hs-cTnT group serving as a reference, the adjusted hazard ratio was 5.0 (95% confidence interval 1.0–23.8; *P* = 0.046) for the CAD and elevated hs-cTnT group. In addition, the subgroup analysis showed similar findings among the patients without severe CAD.

**Conclusions:**

In patients with HCM, combined evaluation of both CAD and hs-cTnT might facilitate more reliable prediction of MACEs than evaluation of a single marker. These may serve as clinically useful markers to guide risk management.

## Background

Hypertrophic cardiomyopathy (HCM) is the most commonly inherited cardiovascular disease, with a prevalence of 0.2% in the general population. It has a higher prevalence (0.5%) when both clinical and genetic diagnoses are taken into account [1–3]. HCM has been regarded as having a poor prognosis with limited management options. During the last 2 decades, important advances in contemporary management strategies have greatly improved the life expectancy and quality of patients with HCM [3–5].

However, some recent studies have suggested that other cardiac or non-cardiac comorbidities might have a greater impact on survival than long-standing HCM itself [6, 7]. For example, some studies have shown that coronary artery disease (CAD) is associated with an increase in poor prognosis in patients with HCM [8, 9]. The serum concentration of high-sensitivity cardiac troponin T (hs-cTnT), a sensitive and specific marker of myocardial injury, is also reportedly effective in predicting adverse outcomes in patients with HCM [10, 11]. In general, CAD can cause a decrease in coronary blood flow to different degrees, and in patients who have HCM without myocardial infarction, an elevated hs-cTnT concentration may indicate microvascular dysfunction [10, 12]. These parameters might represent the total ischemic burden of the myocardium in HCM, which is considered to be associated with poor outcomes. However, whether evaluations of CAD and hs-cTnT might supplement each other and thus become more reliable prognostic markers in patients with HCM remains unclear. The present study was therefore performed to examine the efficiency of combined evaluation of these two markers for prediction of adverse events in patients with HCM.

## Methods

### Study population

**Fig. 1:**
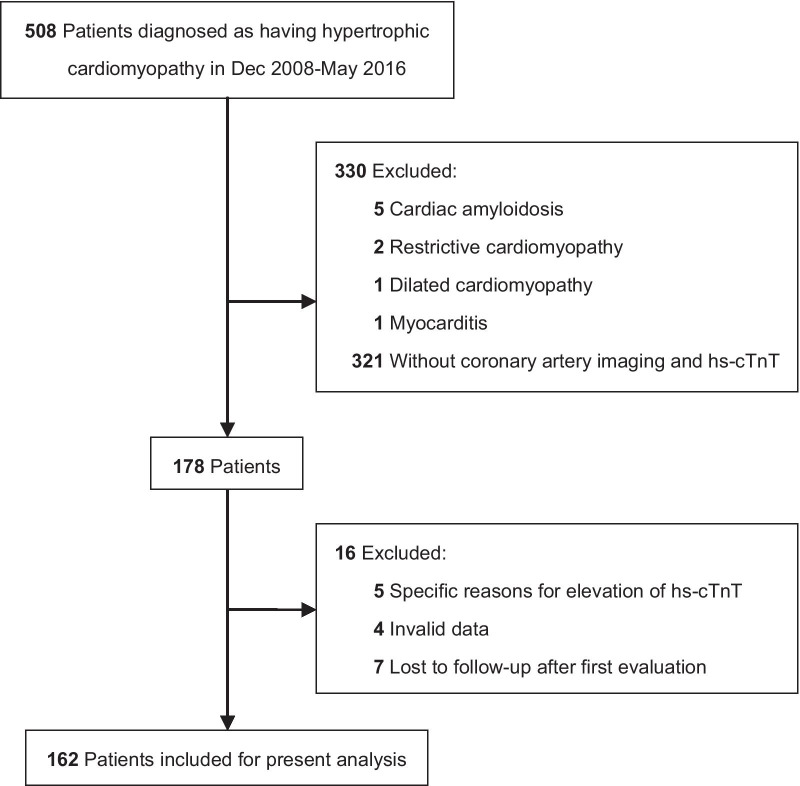
Study flow diagram

This retrospective, single-center, longitudinal study was performed at West China Hospital of Sichuan University (a tertiary referral center in Chengdu, China). From December 2008 to May 2016, 508 consecutive inpatients with a diagnosis of HCM were enrolled in the study. The diagnosis of HCM was based on a wall thickness of ≥ 15 mm in one or more left ventricular (LV) myocardial segments as measured by transthoracic echocardiography or cardiac magnetic resonance imaging that could not be solely explained by abnormal loading conditions [1]. The patients’ data were input twice by medical professionals. If any inconsistency was encountered, the data were rechecked. Patients who were diagnosed with inherited metabolic diseases or syndromic causes of HCM were excluded from the study. Other detailed information were reported in a recently published study [13]. The final study population comprised 162 patients (Fig. 1), none of whom had a history of coronary stent implantation before the first evaluation.

The study was approved by the Ethics Committee on Medical Research of West China Hospital of Sichuan University and conformed to the principles of the Helsinki declaration. The requirement for informed consent was waived because of the retrospective nature of the study.

### Clinical evaluation

Evaluation of patients included a medical history, clinical examination, and Doppler echocardiography. All patients underwent a two-dimensional transthoracic echocardiography examination conducted by a single experienced echocardiographer. LV hypertrophy, LV end-diastolic dimension, LV ejection fraction, LV outflow tract (LVOT) pressure gradient, and left atrial (LA) diameter were assessed by standard techniques. Maximum LV wall thickness was defined as the greatest thickness in any single segment, and LVOT obstruction was defined as a peak LVOT gradient of ≥ 30 mmHg in the resting state [1, 9].

### Evaluation of CAD and hs-cTnT

The severity of CAD was assessed by coronary angiography (n = 129) or coronary computed tomography (CT) angiography (n = 33). Severe CAD was defined as a single luminal diameter stenosis of ≥ 50% in the left main coronary artery or ≥ 70% in other major epicardial branches or the presence of two luminal diameter stenoses of ≥ 50%. Mild to moderate CAD was defined as luminal diameter stenoses that did not meet the criteria for severe CAD [8]. In addition, patients who agreed to undergo percutaneous coronary intervention (PCI) at the baseline evaluation were also defined as having severe CAD.

CAD was severe in 10 patients, and 5 agreed to undergo PCI at baseline. CAD was mild to moderate in 45 patients, and none of these patients agreed to undergo PCI. Because of the small number of patients with severe CAD, we combined these patients with those who had mild to moderate CAD to form a new group (CAD group, n = 55). A total of 107 patients had normal coronary arteries (NCA); among them, 0 had left main disease (≥ 50%), 8 had proximal lesions of the left anterior descending branch (≥ 50%), 2 had right coronary artery disease (≥ 50%), and 3 had left circumflex artery disease (≥ 50%). 5 in one vessels with luminal stenosis ≥ 50%, 4 in two vessels with luminal stenosis ≥ 50%, and 1 in three vessels with luminal stenosis ≥ 50%. The hs-cTnT concentration was assayed using electrochemiluminescence according to the manufacturer’s instructions (Roche Diagnostics, Mannheim, Germany). The reference range of hs-cTnT in an apparently healthy adult population is ≤ 14 ng/L (99th percentile) [14]. Seventy-seven patients had an hs-cTnT concentration within the reference range, and 85 had an elevated hs-cTnT concentration.

### Follow-up evaluation

Follow-up was carried out via medical records and telephone contact with patients themselves or their referring relatives. All patients were followed from the first evaluation to the endpoint or the most recent evaluation.

The primary endpoint of this study was any major adverse cardiovascular events (MACEs), defined as a composite of cardiovascular death, a thromboembolic event, and myocardial infarction/PCI. Cardiovascular death was defined as mortality resulting from any cardiovascular disease, including sudden cardiac death, heart failure-related death, myocardial infarction-related death, perioperative cardiac death, or appropriate shock from an implantable cardioverter defibrillator that was equal to sudden cardiac death. Thromboembolic events included ischemic stroke, transient ischemic attack, and peripheral embolism.

### Statistical analysis

Descriptive statistics (mean ± standard deviation, percentage, etc.) were used to summarize the baseline characteristics. Differences in continuous variables were assessed using one-way analysis of variance, and categorical variables were compared by the chi-square test. The patients were divided into four new groups to assess the associations of the two markers with the primary endpoint: those with NCA and a normal hs-cTnT concentration, those with CAD and a normal hs-cTnT concentration, those with NCA and an elevated hs-cTnT concentration, and those with CAD and an elevated hs-cTnT concentration. MACEs were graphically represented using Kaplan–Meier curves, and the log rank test was used for comparison. Baseline variables that were considered clinically relevant or that showed a univariate relationship with the outcome (*P* < 0.1) were entered into a multivariate Cox proportional hazards regression model. Variables for inclusion were carefully chosen, given the number of events available, to ensure parsimony of the final model. Additionally, we fitted a multivariable model as a sensitivity analysis using Lasso regression, a penalized regression method suitable for data sets with few events [15]. We used Lasso regression to select the potential variables on the basis of lambda.min and constructed a multivariate Cox model. Moreover, we reported the net reclassification index with hs-cTnT in addition to the presence of CAD.

All analyses were performed with R version 3.6.3, including the “compareGroups,” “rms,” “survminer,” “tidyverse,” “glmnet,” “nricens,” and “base” packages (http://www.R-project.org). All tests were two-sided, and P values of < 0.05 were considered statistically significant.

## Results

### Baseline characteristics

Compared with the patients in the NCA group, the patients in the CAD group were older and had higher incidences of hypertension, diabetes, current or prior tobacco use, vascular diseases, and LVOT obstruction. In addition, the CAD group had more prescriptions of aspirin, clopidogrel, and statins. The patients in the elevated hs-cTnT group were older and more commonly had a history of vascular disease and New York Heart Association (NYHA) class III/IV heart disease than the patients in the normal hs-cTnT group. The patients in the elevated hs-cTnT group had a larger LA diameter and maximum LV wall thickness, a higher high-density lipoprotein cholesterol concentration, and a lower LV ejection fraction (detailed data not shown).

According to the four newly defined groups mentioned above, the NCA and normal hs-cTnT group comprised 54 patients, the CAD and normal hs-cTnT group comprised 23 patients, the NCA and elevated hs-cTnT group comprised 53 patients, and the CAD and elevated hs-cTnT group comprised 32 patients. The differences among the four groups are presented in Table 1.

**Table 1:** Baseline characteristics

Variables	All patients (n = 162)	NCA, normal hs-cTnT (n = 54)	CAD, normal hs-cTnT (n = 23)	NCA, elevated hs-cTnT (n = 53)	CAD, elevated hs-cTnT (n = 32)	*P* value
Age (years)	57.5 ± 13.5	52.2 ± 12.5	63.3 ± 11.5	55.9 ± 14.4	64.6 ± 10.1	< 0.001
Gender, male	95 (58.6%)	31 (57.4%)	16 (69.6%)	30 (56.6%)	18 (56.2%)	0.722
Family history of HCM	10 (6.2%)	3 (5.6%)	1 (4.3%)	4 (7.5%)	2 (6.2%)	0.952
Family history of SCD	4 (2.5%)	1 (1.9%)	1 (4.3%)	2 (3.8%)	0 (0.0%)	0.658
Current or prior tobacco use	60 (37.0%)	18 (33.3%)	13 (56.5%)	15 (28.3%)	14 (43.8%)	0.093
NYHA class III/IV	52 (32.1%)	10 (18.5%)	7 (30.4%)	22 (41.5%)	13 (40.6%)	0.050
*Symptoms*						
Chest pain	121 (74.7%)	41 (75.9%)	18 (78.3%)	35 (66.0%)	27 (84.4%)	0.274
Palpitations	64 (39.5%)	22 (40.7%)	8 (34.8%)	23 (43.4%)	11 (34.4%)	0.816
Syncope/pre-syncope	47 (29.0%)	16 (29.6%)	7 (30.4%)	20 (37.7%)	4 (12.5%)	0.101
Dyspnea	98 (60.5%)	29 (53.7%)	13 (56.5%)	34 (64.2%)	22 (68.8%)	0.493
*Comorbidities*						
Atrial fibrillation	18 (11.1%)	4 (7.4%)	4 (17.4%)	5 (9.4%)	5 (15.6%)	0.479
Hypertension	64 (39.5%)	14 (25.9%)	14 (60.9%)	15 (28.3%)	21 (65.6%)	< 0.001
Diabetes	15 (9.3%)	4 (7.4%)	3 (13.0%)	3 (5.7%)	5 (15.6%)	0.396
Vascular diseases	18 (11.1%)	1 (1.9%)	0 (0.0%)	5 (9.4%)	12 (37.5%)	< 0.001
*Therapies*						
Aspirin	38 (23.5%)	8 (14.8%)	8 (34.8%)	6 (11.3%)	16 (50.0%)	< 0.001
Clopidogrel	14 (8.6%)	2 (3.7%)	1 (4.3%)	3 (5.7%)	8 (25.0%)	0.003
Warfarin	8 (4.9%)	2 (3.7%)	0 (0.0%)	3 (5.7%)	3 (9.4%)	0.428
Statins	69 (42.6%)	15 (27.8%)	15 (65.2%)	15 (28.3%)	24 (75.0%)	< 0.001
Beta-blocker	131 (80.9%)	44 (81.5%)	21 (91.3%)	42 (79.2%)	24 (75.0%)	0.487
Diltiazem	10 (6.2%)	4 (7.4%)	2 (8.7%)	2 (3.8%)	2 (6.2%)	0.820
ICD	7 (4.3%)	1 (1.9%)	0 (0.0%)	5 (9.4%)	1 (3.1%)	0.446
Pacemaker	4 (2.5%)	1 (1.9%)	1 (4.3%)	1 (1.9%)	1 (3.1%)	
Septal myectomy	1 (0.6%)	0 (0.0%)	0 (0.0%)	1 (1.9%)	0 (0.0%)	0.293
Alcohol septal ablation	28 (17.3%)	14 (25.9%)	2 (8.7%)	6 (11.3%)	6 (18.8%)	
*Echocardiography data*						
LVEDD (mm)	44.4 ± 5.6	43.9 ± 5.1	44.7 ± 4.8	44.6 ± 6.9	44.7 ± 4.5	0.883
LA diameter (mm)	40.7 ± 6.8	38.8 ± 6.3	40.4 ± 7.2	42.3 ± 7.3	41.3 ± 5.9	0.056
MWT (mm)	18.9 ± 4.8	18.6 ± 5.0	17.1 ± 3.1	20.0 ± 5.4	19.1 ± 4.1	0.108
LVEF (%)	67.5 ± 8.6	70.3 ± 4.7	68.9 ± 5.2	65.3 ± 11.9	65.6 ± 7.8	0.008
LVOT obstruction	78 (48.1%)	27 (50.0%)	11 (47.8%)	17 (32.1%)	23 (71.9%)	0.005
*Biochemical markers*						
Hs-cTnT (ng/L)	30.9 ± 40.1	8.8 ± 3.5	9.4 ± 3.0	47.8 ± 43.1	55.5 ± 53.9	< 0.001
LDL-C (mmol/L)	2.5 ± 0.8	2.6 ± 0.8	2.3 ± 0.7	2.4 ± 0.8	2.4 ± 0.8	0.358
HDL-C (mmol/L)	1.3 ± 0.4	1.2 ± 0.3	1.2 ± 0.4	1.4 ± 0.5	1.3 ± 0.5	0.186

Values are mean ± SD or n (%)

*NCA* normal coronary artery, *hs-cTnT* high-sensitivity cardiac troponin T, *CAD* coronary artery disease, *HCM* hypertrophic cardiomyopathy, *SCD* sudden cardiac death, *ICD* implantable cardioverter defibrillator, *LVEDD* left ventricular end-diastolic dimension, *LA* left atrial, *MWT* maximal left ventricular wall thickness, *LVEF* left ventricular ejection fraction, *LVOT* left ventricular outflow tract, *LDL-C* low density lipoprotein cholesterin, *HDL-C* high density lipoprotein cholesterin

### Follow-up data

**Fig. 2:**
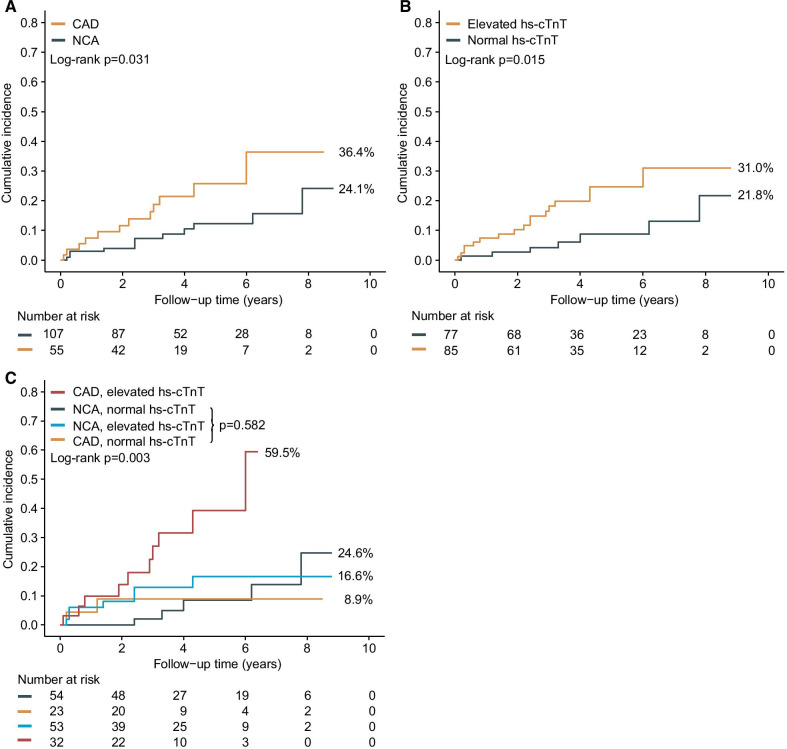
Cumulative incidence of major adverse cardiovascular events. **A** Cumulative incidence of MACEs of patients with CAD versus patients with NCA. **B** Cumulative incidence of MACEs of patients with elevated hs-cTnT versus patients with normal hs-cTnT. **C** Cumulative incidence of MACEs in the four groups according to CAD and hs-cTnT. Abbreviations as in Table 1 and 2

The patients were followed for a median period of 3.7 years (interquartile range 2.4–5.6 years; total, 632.3 person-years [PYs]), and 24 (14.8%) reached the primary endpoint. Clearly defined coronary-related mortality and morbidity developed in about 20.8% of events (Table 2). MACEs more frequently occurred in the CAD group than NCA group (log-rank *P* = 0.031) (Fig. 2A; specifically, 12 (21.8%) MACEs occurred in the CAD group and 12 (11.2%) MACEs occurred in the NCA group. The incidence of MACEs per 100 PYs was 6.4 (95% confidence interval [CI] 2.9–10.0) and 2.7 (95% CI 1.2–4.2) for patients with CAD and NCA, respectively.

**Table 2:** Major adverse cardiovascular events

	NCA, normal hs-cTnT (n = 54)	CAD, normal hs-cTnT (n = 23)	NCA, elevated hs-cTnT (n = 53)	CAD, elevated hs-cTnT (n = 32)
Any MACEs	5	2	7	10
Cardiovascular death	0	0	0	0
SCD	0	0	1	1
Shock	0	0	0	0
Congestive heart failure	0	0	2	3
MI	0	0	1	1
Cardiac perioperative death	0	1	0	0
Thrombo-embolic event	0	0	0	0
Stroke	4	1	2	3
TIA	0	0	0	0
Peripheral embolism	0	0	1	0
MI/PCI	1	0	0	2

Values are n

*MACEs* major adverse cardiovascular events, *MI* Myocardial infarction, *TIA* transient ischemic attack, *PCI* percutaneous coronary intervention; other abbreviations as in Table 1

The elevated hs-cTnT group had more MACEs than the normal hs-cTnT group (log-rank *P* = 0.015) (Fig. 2B). Specifically, MACEs occurred in 17 (20.0%) patients in the elevated hs-cTnT group and in 7 (9.1%) patients in the normal hs-cTnT group. The incidence of MACEs was 5.8 (95% CI 3.1–8.4) and 2.1 (95% CI 0.6–3.6) per 100 PYs in the elevated hs-cTnT group and normal hs-cTnT group, respectively.

Among the newly defined groups, the Kaplan–Meier curve showed that the clinical course was significantly worse in the CAD and elevated hs-cTnT group (log-rank *P* = 0.003) (Fig. 2C); there was no difference among the other three groups (log-rank *P* = 0.582) (Fig. 2C). Table 2 shows the specific MACEs in the different groups. MACEs occurred in 5 (9.3%) patients in the NCA and normal hs-cTnT group, 2 (8.7%) patients in the CAD and normal hs-cTnT group, 7 (13.2%) patients in the NCA and elevated hs-cTnT group, and 10 (31.3%) patients in the CAD and elevated hs-cTnT group. The incidence of MACEs was 10.4 (95% CI 4.3–16.6) per 100 PYs in the CAD and elevated hs-cTnT group, which was much higher than that in the other three groups (Table 3).

**Table 3:** Associations of both coronary artery disease and high-sensitivity cardiac troponin T with major adverse cardiovascular events

	NCA, normal hs-cTnT (n = 54)	CAD, normal hs-cTnT (n = 23)	NCA, elevated hs-cTnT (n = 53)	CAD, elevated hs-cTnT (n = 32)
MACEs (n)	5	2	7	10
Person-years	246.8	90.4	199.3	95.8
Incidence rate (95% CI)^a^	2.0 (0.3–3.8)	2.2 (0.0–5.2)	3.5 (1.0–6.1)	10.4 (4.3–16.6)
Unadjusted HR (95% CI), P	1	1.1 (0.2–5.7), 0.908	1.9 (0.6–6.0), 0.287	5.5 (1.8–16.8), 0.003
*Adjusted HR (95% CI), P*				
Model 1	1	0.6 (0.1–3.3), 0.526	1.6 (0.5–5.2), 0.436	3.4 (1.0–11.1), 0.044
Model 2	1	0.5 (0.1–3.4), 0.521	1.6 (0.5–5.1), 0.461	3.7 (1.0–13.1), 0.046
Model 3	1	0.6 (0.1–4.6), 0.664	1.9 (0.5–7.1), 0.322	5.7 (1.3–24.1), 0.018
Model 4	1	0.5 (0.1–3.9), 0.542	1.2 (0.3–5.2), 0.765	Model 1 with adjustment for age and gender5.0 (1.0–23.8), 0.046

Model 1 with adjustment for age and gender

Model 2 with adjustment for model 1 plus symptoms and comorbidities, including chest pain, palpitations, syncope/pre-syncope, dyspnea, hypertension, diabetes and atrial fibrillation

Model 3 with adjustment for model 2 plus devices, procedures and medications, including aspirin, warfarin, statins and beta-blocker

Model 4 with adjustment for model 3 plus echocardiographic parameters, including LVEDD, LA diameter, MWT, LVEF and LVOT obstruction

*CI* confidence interval, *HR* hazard ratio; other abbreviations as in Tables 1 and 2

^a^Per 100 person-years

### Univariate and multivariate analyses

The univariate analysis showed seven variables with a *P* value of < 0.1: age, CAD, hs-cTnT, combined evaluation, LA diameter, the use of statins, and the use of beta-blockers.

The multivariate analysis showed that CAD and hs-cTnT tended to be positively associated with MACEs. With the NCA or normal hs-cTnT group as a reference, the adjusted hazard ratios (HRs) were 1.7 (95% CI 0.5–5.6; *P* = 0.371) and 2.8 (95% CI 1.0–8.1; *P* = 0.056) for the CAD or elevated hs-cTnT group, respectively.

The multivariate analysis indicated that combined evaluation of both CAD and hs-cTnT was positively associated with MACEs, which further improved the prognostic values. With the NCA and normal hs-cTnT group as reference, the adjusted HRs were 0.5 (95% CI 0.1–3.9; *P* = 0.542) for the CAD and normal hs-cTnT group, 1.2 (95% CI 0.3–5.2; *P* = 0.765) for the NCA and elevated hs-cTnT group, and 5.0 (95% CI 1.0–23.8; *P* = 0.046) for the CAD and elevated hs-cTnT group (Table 3). Moreover, the multivariate analysis also suggested that the LA diameter (per 1-mm increase; HR 1.066; 95% CI 0.997–1.139; *P* = 0.054), age (per 1-year increase; HR 1.050; 95% CI 0.996–1.106; *P* = 0.069), and use of aspirin (HR 0.273; 95% CI 0.069–1.081; *P* = 0.068) tended to be predictors of MACEs. However, these results should be interpreted with caution because the small events-per-variable ratio meant that the effect of overfitting might be pronounced. As a sensitivity analysis, the Lasso–Cox regression model included five variables: sex, gender, devices, beta-blocker use, and LA diameter. The results showed that the CAD and elevated hs-cTnT group still tended to be positively associated with new-onset MACEs. With the NCA and normal hs-cTnT group serving as the reference, the adjusted HR was 2.8 (95% CI 1.0–10.2; *P* = 0.048) for the CAD and elevated hs-cTnT group. In addition, the data showed that combined CAD could predict MACEs; thus, the net reclassification index with hs-cTnT was further evaluated. The patients have been followed up as long as 8.8 years, according to statistics from the previous study [8], which is also confirmed in our research, showed that the 8-year accumulated incidence of events in patients who have HCM without coronary heart disease is about 20%. With coronary heart disease, however, the incidence of events is 35%.Therefore, the tangents of 0.2 and 0.4 were selected as low, medium, and high risk. The results of the analysis indicated that the addition of hs-cTnT increased the degree of prediction by 15.3%.

### Additional analysis

Patients with severe CAD were recognized to have a poor prognosis, and some of them agreed to undergo PCI at the baseline evaluation. Therefore, we excluded the patients with severe CAD (n = 10), and the remaining 152 patients were included in the subgroup analysis to determine the usefulness of combined evaluation in patients without severe CAD.

The subgroup analysis indicated that 23 MACEs (15.1%) had occurred during a follow-up period of 601.2 PYs (median 3.7 years; interquartile range 2.5–5.7 years). MACEs occurred in 5 (9.3%, 5/54) patients in the NCA and normal hs-cTnT group, 2 (8.7%, 2/23) patients in the CAD and normal hs-cTnT group, 7 (13.2%, 7/53) patients in the NCA and elevated hs-cTnT group, and 9 (40.9%, 9/22) patients in the CAD and elevated hs-cTnT group. The incidence was 13.9 (95% CI 5.5–22.2) per 100 PYs in the CAD and elevated hs-cTnT group, which was much higher than that in the other three groups (range 2.0–3.5 per 100 PYs), and the clinical course was significantly worse in the CAD and elevated hs-cTnT group (log-rank *P* < 0.001). With the NCA and normal hs-cTnT group as a reference, the multivariate analysis suggested that the adjusted HR was 10.7 (95% CI 1.7–66.8; *P* = 0.011) for the CAD and elevated hs-cTnT group.

Decompensated heart failure (NYHA class III/IV) may increase the release of troponin and is associated with an increased risk of death. Therefore, we conducted a sensitivity analysis restricted to patients without symptoms/signs of heart failure. Fifty-two patients had NYHA class III/IV heart failure; after excluding these patients, 110 patients were involved in the sensitivity analysis, and 15 of these patients developed MACEs. Control with the normal group, the univariate Cox analysis suggested that for patients with abnormal troponin and coronary heart disease, the HR was 5.0 (95% CI 1.4–17.7; *P* = 0.013). However, no further multivariate analysis was performed because of the low number of events.

## Discussion

We assessed the usefulness of combined evaluation of both CAD and hs-cTnT for prediction of MACEs in patients with HCM. We found that combined evaluation of these two markers is a more reliable predictor of MACEs than evaluation of a single marker. Thus, our findings might be of value to classify the prognosis and guide risk management in patients with HCM.

Many studies have shown that patients with HCM have a relatively benign prognosis and a mortality rate similar to that in the general population [3–5]; however, other comorbidities might pose a greater threat to survival than long-standing HCM itself [6, 7, 9]. As an important comorbidity, CAD can predict future adverse outcomes in patients with HCM. Sorajja et al. [8] showed that patients with concomitant severe CAD had an increased risk of death. In a recent study, Shin et al. [9] reported that severe CAD served as an independent predictor of adverse cardiovascular events in patients with HCM, and the incidence of clear CAD-related mortality or morbidity was about 25% among the events. In our study, the clinical course was significantly worse in the CAD group, and the incidence of clear CAD-related MACEs was about 20.8% among the events. Therefore, the evaluation of CAD may be important in patients with HCM. Moreover, elevated troponin is commonly seen in patients with HCM, and an elevated hs-cTnT above a cut-off point of 14 ng/l is seen in 26% to 54% of patients with HCM [11]. The hs-cTnT concentration has been positively related to the LV wall thickness [10, 16–18], hemodynamic parameters [10, 16, 17], clinical symptoms [1, 10, 11, 19], and outcomes [1, 10, 11, 19] in patients with HCM. Furthermore, the risk of adverse cardiovascular events seems to be greater with increased degrees of abnormality in the hs-cTnT concentration. Our study also showed similar results; therefore, hs-cTnT is expected to be useful in the clinical evaluation of patients with HCM. Although our multivariate analysis showed that statistical significance was not achieved, our findings suggested that both CAD and elevated hs-cTnT tended to be positively associated with MACEs. We believe that this might have been caused by the relatively small sample and that our results could be improved by a larger sample. Notably, however, the combined evaluation of the two markers significantly improved the predictive values. This combined evaluation achieved statistical significance in the same sample and therefore indicates the clinical usefulness of combined evaluation.

In the present study, combined evaluation of both CAD and hs-cTnT had better predictive value than evaluation of a single marker. When patients with HCM have CAD, the coronary blood flow might decrease to different degrees, which could cause MACEs. Furthermore, patients with HCM who develop CAD usually have more cardiovascular risk factors [8, 20]. From this viewpoint, our study supports previous findings. These cardiovascular risk factors can exacerbate the structural abnormalities and inherent endothelial dysfunction in patients with HCM [8, 21], further increasing the detrimental impact on the prognosis. However, the mechanisms underlying the release of hs-cTnT in patients with HCM remain unclear. It is speculated that the increase may be caused by relative myocardial ischemia resulting from an imbalance between inappropriate hypertrophy of the myocardium and insufficient coronary arterial supply [10, 12]. In this respect, the present study proposes microvascular dysfunction as an important explanation [12]. Microvascular dysfunction associated with elevated hs-cTnT can result in myocyte injury and subsequent fibrosis in HCM, leading to poor outcomes [10, 12]. Given the increased myocardial mass and high myocardial oxygen demand, patients with microvascular dysfunction might be particularly susceptible to the additional ischemic burden of CAD [22], further increasing the incidence of adverse outcomes. As mentioned above, epicardial CAD might respect a diminished coronary flow and a cluster of cardiovascular risk factors, whereas elevated hs-cTnT could respect myocardial injury and microvascular dysfunction; these might represent the total ischemic burden of the myocardium. Therefore, combined evaluation of both CAD and hs-cTnT should have greater prediction ability than evaluation of a single marker. Notably, performing coronary artery imaging in patients with HCM who have both elevated hs-cTnT and risk factors for CAD is taken for granted. Therefore, we should pay more attention to patients with either elevated hs-cTnT or risk factors for CAD, especially asymptomatic patients; such patients may need further coronary artery imaging.

This study has several limitations. First, this was a single-center retrospective clinical study and therefore may have certain inherent biases. We could only use the patients’ existing data. In total, 321 patients were excluded because of a lack of coronary artery imaging and hs-cTnT data, which may represent a selection bias. Although this was a specially selected group of patients, it still indicates the risk of CAD combination for this type of patient. Additionally, some patients’ baseline data excluded renal function parameters. However, renal insufficiency was not diagnosed in the medical records; therefore, we can partially rule out renal insufficiency as a cause of the rise in troponin. These limitations warrant prospective studies. Second, coronary imaging and hs-cTnT measurement should ideally have been performed during a steady state. In our study, 52 (32.1%) patients had NYHA class III/IV heart failure, which may suggest the acute phase of disease. Therefore, larger prospective studies are needed to confirm and extend the present findings. Third, the number of MACEs was so small (n = 24) that the subanalysis and grouping analysis may have limited the statistical value; however, these analyses still provided useful findings. Although the multivariate analysis showed that statistical significance was not achieved, our findings suggest that both CAD and elevated hs-cTnT tended to be positively associated with MACEs. We believe that this could be improved by a larger sample. Because of the small number of events, the role of combined evaluation of CAD and hs-cTnT in specific subgroups of patients with HCM remains to be further evaluated. Fourth, not all patients agreed to undergo coronary angiography, and 33 (20.4%) patients underwent coronary CT angiography; this might suggest that the study population was somewhat heterogeneous. However, coronary CT angiography has high negative and positive predictive values in patients with HCM [22], and the use of coronary CT angiography should be acceptable. Fifth, because the severity of CAD was assessed by coronary angiography or coronary CT angiography without intravascular ultrasound or optical coherence tomography, the specific causes of stenosis cannot be determined. However, the performance of intravascular ultrasound and optical coherence tomography for all patients was not feasible for pragmatic and logistical reasons. Finally, severe CAD usually requires intervention and has a very poor prognosis. Therefore, the combination of patients with severe CAD and mild to moderate CAD into a single group may have caused bias. However, the additional analysis indicated similar results, further supporting the main analysis.

## Conclusions

Combined evaluation of both CAD and hs-cTnT could be a more reliable predictor of MACEs than evaluation of a single marker in patients with HCM. The present study findings may be of value to classify the prognosis and guide risk management in patients with HCM. Larger studies are needed to confirm these findings.

## Data Availability

The datasets used and/or analysed during the current study are available from the corresponding author on reasonable request.

## Abbreviations

CAD: Coronary artery disease
hs-cTnT: High-sensitivity cardiac troponin T
MACEs: Major adverse cardiovascular events
HCM: Hypertrophic cardiomyopathy
PYs: Person-years
NCA: Normal coronary artery
LV: Left ventricular
LVEDD: LV end-diastolic dimension
LVEF: LV ejection fraction
LVOT: LV outflow tract
LA: Left atrial
MWT: Maximum LV wall thickness
PCI: Percutaneous coronary intervention
SCD: Sudden cardiac death
ICD: Implantable cardioverterdefibrillator
